# Chicken caspase-3 promotes IBDV replication via the cleavage of IRF7

**DOI:** 10.3389/fmicb.2026.1741783

**Published:** 2026-02-23

**Authors:** Yang Chen, Jinnan Chen, Yanhua Xiang, Simin Wei, Minhui Zhao, Kexuan Fu, Yihai Li, Hongjun Chen, Ping Wei, Xiumiao He

**Affiliations:** 1 Guangxi Key Laboratory for Polysaccharide Materials and Modifications, School of Marine Sciences and Biotechnology, Guangxi Minzu University, Nanning, Guangxi, China; 2 College of Animal Science and Technology, Guangxi University, Nanning, Guangxi, China; 3 College of Veterinary Medicine, China Agricultural University, Beijing, China

**Keywords:** apoptosis, caspase-3, infectious bursal disease virus (IBDV), innate immunity, IRF7, viral replication

## Abstract

Infectious bursal disease virus (IBDV) is a highly contagious pathogen that causes severe immunosuppression in chickens, leading to significant economic losses. While apoptosis is a critical host defense mechanism, many viruses exploit it to enhance replication. Here, we demonstrate that IBDV infection induces caspase-dependent apoptosis and that the executioner caspase, Caspase-3, is activated to promote viral replication. Mechanistically, we identify a novel immune evasion strategy: Caspase-3 directly cleaves and degrades interferon regulatory factor 7 (IRF7), a key transcriptional activator of the type I interferon (IFN-β) pathway. This cleavage potently suppresses the host antiviral innate immune response. Furthermore, Caspase-3 activity exacerbates IBDV-induced apoptosis. Consequently, pharmacological inhibition of Caspase-3 significantly reduced viral load and apoptosis, whereas its overexpression produced opposite effects. Our findings reveal that IBDV hijacks the host apoptotic effector Caspase-3 to dismantle the IRF7-mediated antiviral defense, thereby facilitating viral immune evasion and replication. This study provides new insights into viral pathogenesis and suggests Caspase-3/IRF7 as potential therapeutic targets for IBDV control.

## 1 Introduction

Infectious bursal disease virus (IBDV) is a highly contagious pathogen that predominantly affects young chickens, causing significant immunosuppression through the destruction of B lymphocytes within the bursa of Fabricius (Dey et al., 2019; Eterradossi and Saif, 2020). This immunosuppression compromises the host’s ability to mount an effective immune response, leading to increased susceptibility to other infectious agents, and ultimately, severe economic losses in the poultry industry (Berg, 2000; Kibenge et al., 1988). IBDV is classified into two serotypes, serotype 1 and serotype 2. Research has shown that only serotype 1 is pathogenic to chickens. Serotype 1 can be further divided into five phenotypes, including classical IBDV (cIBDV) (Cosgrove, 1962), very virulent IBDV(vvIBDV) (Chettle et al., 1989), attenuated IBDV(attIBDV) (Winterfield et al., 1981), antigenic variant IBDV(avIBDV) (Jackwood and Saif, 1987), and recently identified Chinese novel variant IBDV (nvIBDV) (Fan et al., 2019; Wang et al., 2022b). Despite the availability of vaccines, IBDV remains a pervasive challenge, as new viral strains continually emerge, and vaccine efficacy varies. Understanding the molecular mechanisms of IBDV pathogenesis and host immune evasion is thus crucial for developing more effective control strategies.

Viral infection triggers systemic immune responses in host cells via pattern-recognition receptors (PRRs) and adaptor protein pairs that detect the pathogen-associated molecular patterns (PAMPs) and transmit downstream signals (Goubau et al., 2013). These receptor-adaptor pairs lead to the activation of different transcriptional factors such as nuclear factor kB (NF-kB) and IRF3/IRF7, and the production of various cytokines, including type I interferons (I-IFNs) (McFadden et al., 2017). Released IFNs bind to interferon receptors (IFNARs) and induce the expression of numerous IFN-stimulated genes (ISGs) (Stark and Darnell, 2012), which interrupt almost every stage of the viral life cycle (Skaug and Chen, 2010), culminating in the establishment of an antiviral state. Studies revealed that IRF3 is genetically deficient in chickens and other avian species (Cheng et al., 2019; Cormican et al., 2009). Chicken IRF7 (chIRF7) is involved in both chicken STING (chSTING)- and chicken MAVS (chMAVS)-mediated IFN-β regulation in response to DNA and RNA viral infections, respectively (Cheng et al., 2019; Kim and Zhou, 2015).

To prevent viruses from subverting cellular machinery for their own replication, both DNA and RNA viruses cause infected cells to undergo apoptosis (Best, 2008; Yatim and Albert, 2011), the best-known mechanism of programmed cell death. Apoptosis is mediated through the activation of apoptotic caspases. Based on their physiological functions, caspases are classified as either inflammatory or apoptotic. Inflammatory caspases (caspase-1, -4, -5, and -12) participate in inflammasome activation and induce inflammatory cell death, called pyroptosis (Lamkanfi and Dixit, 2014; Wang et al., 2017), while apoptotic caspases (caspase-2, -3, -6, -7, -8, -9, and -10) mediate apoptotic activation (Kumar, 2007; McIlwain et al., 2013). Apoptosis is initiated by two pathways: intrinsic and extrinsic. The extrinsic pathway is activated by the engagement of transmembrane receptors by its ligands, eventually leading to the activation of caspase-8 and its downstream effector caspases. Intrinsic apoptosis triggers the activation of pro-apoptotic members of the Bcl2 family, which controls the Bax/Bak channel and induces mitochondrial outer membrane permeabilization (MOMP) (Youle and Strasser, 2008). MOMP results in the release of mitochondrial factors into the cytosol, including cytochrome c, which binds to Apaf-1 and activates caspase-9, and in turn the downstream effector caspases -3, -6, and -7 (Riedl and Salvesen, 2007). Liu and Vakharia (2006) found that IBDV caused significant cell apoptosis and related factors such as Caspase-3, -8, and -9 produced changes in response. Therefore, apoptotic caspases are speculated to play an important role in the pathogenic mechanism of IBDV.

This study investigates the role and mechanistic basis of Caspase-3 in IBDV infection. Here, we demonstrate that Caspase-3 is crucial for controlling IRF7 pathway in IBDV-infected cells. We found that IBDV causes significant cell death and apoptosis on DF-1 cells, while altering the expression of Caspase-3, a key factor in apoptosis. Notably, Caspase-3 markedly enhances IBDV replication by facilitating the targeted degradation of IRF7. Our findings unveil Caspase-3 as a pivotal regulator of IBDV-induced innate immune responses and provide molecular insights into the interplay between viral replication and host immune evasion.

## 2 Materials and methods

### 2.1 Viruses, cells, and antibodies

The vvIBDV strain used in this study was NN1172 strain which was isolated and identified by our group (He et al., 2014). The DF-1 chicken fibroblast cell line was obtained from Pricella. Dulbecco’s modified Eagle’s medium (DMEM) (12100061; Gibco, United States) supplemented with 10% fetal bovine serum (FBS) (12483020; Gibco, United States) was used to culture chicken fibroblast cell line DF-1 cells. Anti-IBDV VP2 mouse monoclonal antibody (mAb) was prepared in our laboratory. Other antibodies used in our study were anti-IRF7 rabbit polyclonal antibody (pAb) (bs-2994R, BIOSS, China), anti-Caspase-3 (Pro and Active) mouse mAb (31A1067, Novus, United States), anti-β-actin mouse mAb (CW0096M, CWBIO, China), anti-His mouse mAb (M20001, Abmart, China), anti-HA mouse mAb (M20003, Abmart, China), goat anti-rabbit IgG H&L antibody (CW0103S, CWBIO, China), goat anti-mouse IgG H&L antibody (CW0102S, CWBIO, China), AbBox Fluor 594-labeled goat anti-rabbit IgG (BD9279, Biodragon, China), FITC-labeled goat anti-mouse IgG (BF05001, Biodragon, China). Other reagents were the caspase inhibitor Z-VAD (HY-16658B, MedChemExpress, United States), Caspase-3 inhibitor Z-DEVD (HY-12466, MedChemExpress, United States), SYBR Green qPCR Mix (11201ES, YEASEN, China), Cell Counting Kit-8 (C6005, NCM Biotech, China), Annexin V-FITC Apoptosis Detection Kit (556547, BD Biosciences, United States), Lipo8000 transfection reagent (C0533, Beyotime, China), RIPA buffer (P0013B, Beyotime, China), PBS (C0221A, Beyotime, China), Protease Inhibitor Cocktail (CW2200S, CWBIO, China), 5 × SDS loading buffer (CW0027S, CWBIO, China), TRIzon Reagent (CW0580, CWBIO, China), HiFiScript gDNA Removal RT MasterMix (CW2020M, CWBIO, China), protein A/G agarose (80104G, Invivogen, FR).

### 2.2 IBDV infection

DF-1 cells were seeded in 24-well plates at a density of 1.2 × 10^5^ cells per well and cultured until reaching 90% confluence. The growth medium was aspirated, and cell monolayers were washed twice with PBS. Subsequently, each well was inoculated with 100 μL of serially diluted IBDV suspended in DMEM without FBS. After incubation at 37°C for 1 h to allow viral adsorption, the inoculum was removed, and cells were gently washed twice with PBS to eliminate unbound virions. The infected monolayers were then maintained in DMEM supplemented with 1% FBS (v/v) and incubated at 37°C under 5% CO_2_ condition.

### 2.3 Plasmid construction

Plasmid construction: The coding sequences (CDS) of Caspase-3, IRF7, and IBDV viral proteins (VP1, VP2, VP3, VP4, VP5) were amplified by PCR from relevant cDNA or viral genomic templates using specific primers containing appropriate restriction enzyme sites. The PCR products were digested with the corresponding restriction enzymes and subsequently ligated into the similarly digested mammalian expression vectors, p3 × Flag-CMV-14, pcDNA3.1(+) with an N-terminal HA or His tag, or other vectors as specified. All constructed plasmids were verified by Sanger sequencing (performed by Sangon Biotech, China) to ensure the absence of unintended mutations. The primer sequences and cloning sites are available upon request.

### 2.4 Transfection

DF-1 cells were seeded in 24-well plates at a density of 1.2 × 10^5^ cells per well and cultured until reaching 90% confluence. Transfection was performed using Lipo8000 Transfection Reagent according to the manufacturer’s protocol.

### 2.5 *In vitro* cleavage assay

*In vitro* protease cleavage assays were performed as previously described (Wang et al., 2017). Briefly, recombinant caspase-3 protein was incubated with recombinant IRF7 protein in cleavage buffer (100 mM HEPES, 10% (w/v) sucrose, 0.1% (w/v) CHAPS, pH 7.0, 10 mM DTT) at 37°C for 2 h, followed by immunoblot analysis.

### 2.6 Western blot

Cell samples were harvested and the expression of denatured proteins was analyzed using SDS-PAGE. Cells were lysed with RIPA buffer containing Protease Inhibitor Cocktail. The lysates were mixed with 5 × SDS loading buffer, boiled for 10 min, and separated on 10% SDS-PAGE gels. Proteins were subsequently transferred onto a nitrocellulose membrane. The membrane was then blocked in 5% (w/v) skim milk for 2 h, followed by incubation with monoclonal or polyclonal antibodies for 2 h. After being washed three times (10 min each) with TBST, the membrane was incubated with goat anti-mouse IgG (H+L) antibody for 1 h., the protein blots were finally visualized using the WD-9423BC automatic chemiluminescence imaging system (LIUYI, China) for further analysis.

### 2.7 RT-qPCR

Total RNA was extracted from treated cells using TRIzon Reagent according to the manufacturer’s protocol. RNA concentration and purity were assessed by spectrophotometry (NanoDrop, Thermo Scientific, United States) with acceptance criteria set at A260/A280 ratios of 1.8–2.0. For cDNA synthesis, 1 μg of total RNA was reverse transcribed using HiFiScript gDNA Removal RT MasterMix in a 20 μL reaction volume under the following conditions: 42°C for 15 min (reverse transcription), 85°C for 5 min (enzyme inactivation), followed by immediate cooling to 4°C. Quantitative real-time PCR (qPCR) was performed using the following cycling conditions 95°C for 5 min, 95°C for 10 s, and 60°C for 30 s, followed by 40 cycles. Gene expression levels were calculated using the 2^−ΔΔCt^ method, with β-actin as the endogenous control. For viral load quantification, IBDV copy numbers were determined from CT values using a standard curve generated from serial dilutions of IBDV reference RNA. All reactions were performed in technical triplicates, with negative controls (no-template and no-RT controls) included in each run. Primer sequences are provided in Table 1.

**TABLE 1:** List of primers used in RT-qPCR.

Genes	Direction	Sequence	Product (bp)	Accession no. in GenBank
IBDV	Forward	ACCGGCACCGACAACCTTA	117	FJ615511.1
	Reverse	CCCTGCCTGACCACCACTT		
IRF7	Forward	ACCACATGCAGACAGACTGACAC	146	AF268079
	Reverse	GGAGTGGATGCAAATGCTGCTCTT		
IFN-β	Forward	TTCTCCTGCAACCATCTTC	82	NM001024836.1
	Reverse	GAGGTGGAGCCGTATTCT		
β-actin	Forward	CAACACAGTGCTGTCTGGTGGTA	205	NM_205518.2
	Reverse	ATCGTACTCCTGCTTGCTGATCC		
Caspase-3	Forward	TGGCCCTCTTGAACTGAAAG	139	NM_204725.2
	Reverse	TCCACTGTCTGCTTCAATACC		
Caspase-6	Forward	TCAGAGGAGACAAGTGCCAGAGT	107	NM_001396147.1
	Reverse	TACTGAATCCTGAACGAGAACTGG		
Caspase-7	Forward	CCGAAGTCCTCACTCAGTAACCA	137	XM_046943128.1
	Reverse	TTGCGTGTACCCATTCCTGTT		
Caspase-8	Forward	TGGGAAAGTGGACAAGAGCCT	146	NM_204592.4
	Reverse	CCACAGATGATGCCAGCCAA		
Caspase-9	Forward	CGAAGGAGCAAGCACGACAG	130	XM_046931415.1
	Reverse	CCGCAGCCCTCATCTAGCAT		
Caspase-10	Forward	GCAGCGTTCAGAAGACCACAA	141	XM_040676185.2
	Reverse	CATTGCTTGGCAGTGAAGTAGGT		

### 2.8 CCK8 assay

DF-1 cells were seeded in 96-well plates at a density of 1 × 10^4^ cells/well and cultured in DMEM supplemented with 10% FBS at 37°C under 5% CO_2_ until 90% confluence. The cells were divided into four experimental groups: Control (Untreated cells); IBDV-infected (Cells infected with IBDV); Inhibitor (Cells treated with 50 μM Z-VAD or 20 μM Z-DEVD) and IBDV+inhibitor (Cells pretreated with inhibitors followed by IBDV infection). For the inhibitor and IBDV+inhibitor groups, cells were pretreated with 50 μM Z-VAD or 20 μM Z-DEVD in serum-free DMEM for 2 h at 37°C. After pretreatment, the medium was aspirated, and cells were washed twice with PBS. For IBDV infection, cells in the IBDV-infected and IBDV+inhibitor groups were inoculated with 1 MOI of IBDV in 100 μL serum-free DMEM and incubated for 1 h at 37°C. Unbound virus was removed by PBS washing, and fresh maintenance medium (DMEM with 1% FBS) was added. Inhibitors were reapplied to the respective groups (50 μM Z-VAD or 20 μM Z-DEVD), while control and IBDV-infected groups received 1% maintenance medium. Cells were further incubated for 32 h. After treatment, 10 μL CCK-8 reagent was added to each well, followed by incubation at 37°C for 1.5 h. Absorbance was measured at 450 nm using BioTek Synergy H1 microplate reader (Agilent, United States). Cell viability was calculated as:


$$\left(O\,D_{t\,r\,e\,a\,t\,m\,e\,n\,t}-O\,D_{e\,m\,p\,t\,y}\right)/\left(O\,D_{n\,o\,n\,t\,r\,e\,a\,t\,e\,d}-O\,D_{e\,m\,p\,t\,y}\right)\times 100\%$$

### 2.9 Flow cytometry

DF-1 cells were seeded in 6-well plates and cultured at 37°C with 5% CO_2_ until approximately 80% confluence. Cells were then treated and collected at 12-h post infection (hpi) and 32 hpi. After discarding the supernatant, cells were washed with PBS and digested with trypsin. Digestion was terminated by adding complete culture medium, and the cells were collected into a centrifuge tube. The cells were then centrifuged at 2,000 rpm for 3 min at 4°C. The supernatant was discarded, and the cell pellet was resuspended in pre-chilled PBS, followed by centrifugation at 1,000 rpm for 5 min at 4°C. After discarding the supernatant, the cell pellet was resuspended in 250 μL of 1 × Loading Buffer. The cell count was adjusted to 1 × 10^6^ cells/100 μL for each replicate. Subsequently, 5 μL of Annexin V-FITC and 5 μL of PI were added, and the mixture was gently vortexed and incubated in the dark at room temperature for 15 min. Just before analysis on the flow cytometer, 400 μL of 1 × Binding Buffer was added to each sample.

### 2.10 Co-immunoprecipitation and immunofluorescent staining

Co-Immunoprecipitation (Co-IP) was conducted to determine whether two proteins have interactions. DF-1 cells were co-transfected with the corresponding plasmids. After 48 h post-transfection, Co-IP was performed as follows: cells were washed three times with ice-cold PBS and lysed in 500 mL of RIPA buffer for 30 min. After 12,000 × g centrifugation, the supernatants of cell lysates were incubated with 3 mL anti-Flag mouse mAb or control mouse IgG overnight. Subsequently, 30 mL protein A/G agarose was added to the lysate mixture for 6–8 h. The beads were collected by centrifugation at 3,000 × g for 5 min at 4°C and washed five times with ice-cold PBS.

Immunofluorescent (IF) staining was conducted to determine whether VP3 colocalized with Caspase-3 in the cell. DF-1 cells were co-transfected with p3 × Flag-CMV-14-Caspase-3 and pcDNA3.1-HA-VP3 plasmids. At 36 h after transfection, the cells were fixed with 4% paraformaldehyde for 20 min at room temperature and permeabilized for 15 min with 0.25% Triton X-100. After being blocked with 5% skim milk, cells were incubated with anti-HA mouse mAb and anti-Flag rabbit mAb overnight at 4°C. After being washed three times with PBS, cells were further incubated with FITC-labeled goat anti-mouse IgG and AbBox Fluor 594-labeled goat anti-rabbit IgG secondary antibody at room temperature for 1 h. Cellular nuclei were stained with DAPI for 10 min and viewed with an LAS X laser scanning confocal microscope (Leica, Cologne, Germany).

### 2.11 Statistical analysis

Data are presented as mean ± standard error of the mean (SEM) from at least three independent experiments. The normality of data distribution was assessed using the Shapiro-Wilk test. For comparisons between two groups, statistical significance was determined using a two-tailed, unpaired Student’s *t*-test (for normally distributed data) or the Mann-Whitney U test (for non-normally distributed data). For comparisons among more than two groups, one-way analysis of variance (ANOVA) was performed, followed by the Student-Newman-Keuls (SNK) *post-hoc* test for multiple comparisons when the ANOVA indicated a significant difference (*p* < 0.05). All statistical analyses were performed using GraphPad Prism software (GraphPad Software, United States). A *p*-value of < 0.05 was considered statistically significant (*p < 0.05, **p < 0.01).

## 3 Results

### 3.1 Caspase-dependent cell death and apoptosis induced by IBDV

Caspases play critical roles in multiple viral infections, for example, caspase inhibitors have been reported to prevent cell death caused by the Sindbis virus (Nava et al., 1998). However, it remains unclear whether these inhibitors can reduce the pathogenesis in IBDV-infected cells. To explore the role of apoptotic caspases on the pathogenesis of IBDV, DF-1 cells were treated with the compound Z-VAD or Z-DEVD, and then cells were infected with IBDV, the cell death and apoptosis were evaluated.

**FIGURE 1:**
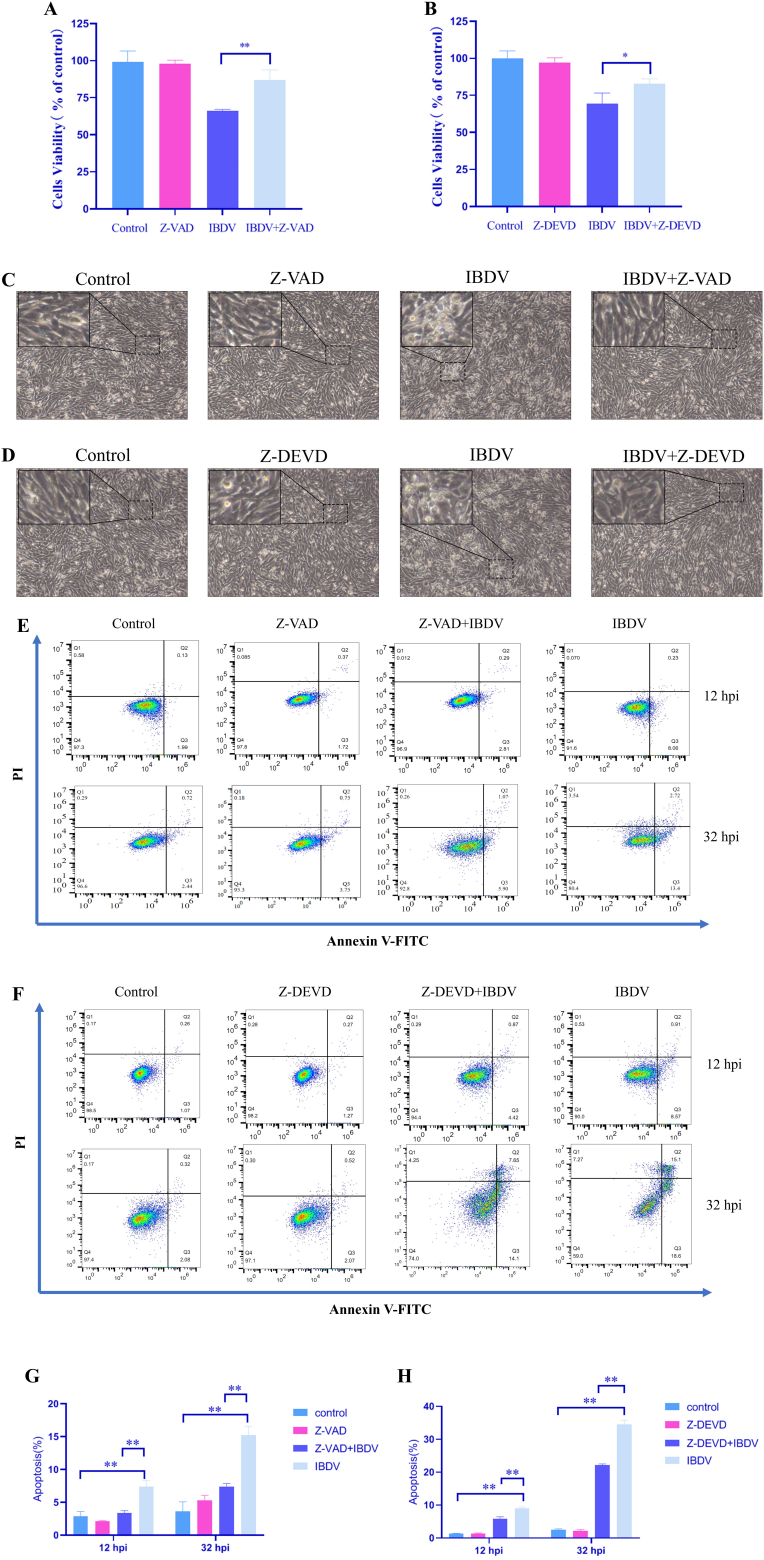
Caspase-dependent cell death and apoptosis induced by IBDV. **(A,B)** The CCK8 assay results show that treatment with Caspase inhibitors improved cell viability in IBDV-infected DF-1 cells, with Z-VAD and Z-DEVD used at concentrations of 50 and 20 μM, respectively. **(C,D)** Caspase inhibitors reduced the cytopathic effect (CPE) induced by IBDV in DF-1 cells, as observed through microscopy (×10). **(E)** Flow cytometry assays of the apoptosis of DF-1 cells in Z-VAD group. **(F)** Flow cytometry assays of the apoptosis of DF-1 cells in Z-DEVD group. **(G,H)** Statistical analysis of the percentage of the Q2 area added to the Q3 area in different treatment. Results are presented as mean ± SD, analyzed using a two-sample Student’s *t*-test. *P < 0.05, **P < 0.01.

Firstly, the cell toxicity of Z-VAD and Z-DEVD were assessed using the CCK8 assay, the result showed that the cell viability in the cell incubated by Z-VAD or Z-DEVD were the same as the control group (Figures 1A,B) which confirmed that both compounds were non-toxic to the cells within the tested concentrations. We then evaluated if there were any effects of the Caspase inhibitors on the cells infected by IBDV. The results showed that treatment with Z-VAD (50 μM) or Z-DEVD (20 μM) significantly improved cell viability evaluated by CCK8 assay (Figures 1A,B) and restored healthy cell morphology as evidenced by less rounded morphology cells and smaller intercellular gaps in cells infected with IBDV and treated by Z-VAD or Z-DEVD as compared by IBDV-infected DF-1 cells (Figures 1C,D).

Given the role of Caspase family members in apoptosis, we further examined the apoptosis rates following treatment with these inhibitors in IBDV infected cells. We employed Annexin-V/PI double staining for detecting apoptosis via flow cytometry. As shown in Figures 1E–H, in the Z-VAD treatment group, the total cell apoptosis rates in Z-VAD+IBDV group were 3.10% at 12 hpi and 6.97% at 32 hpi respectively, which were significantly lower than those from the IBDV- infected control (with 8.29% at 12 hpi and 16.12% at 32 hpi). Similarly, in the Z-DEVD treatment group, the total apoptosis rates in Z-DEVD+IBDV group were 5.29% (12 hpi) and 21.75% (32 hpi), significantly lower than those from the IBDV-infected control (9.48% at 12 hpi and 33.70% at 32 hpi). These results suggested that IBDV-induced cell death/apoptosis is dependent on caspase activation, with caspase-3 in particular might play a critical role in the apoptotic process.

### 3.2 The upregulation of caspase-3 mRNA in IBDV-infected cell

**FIGURE 2:**
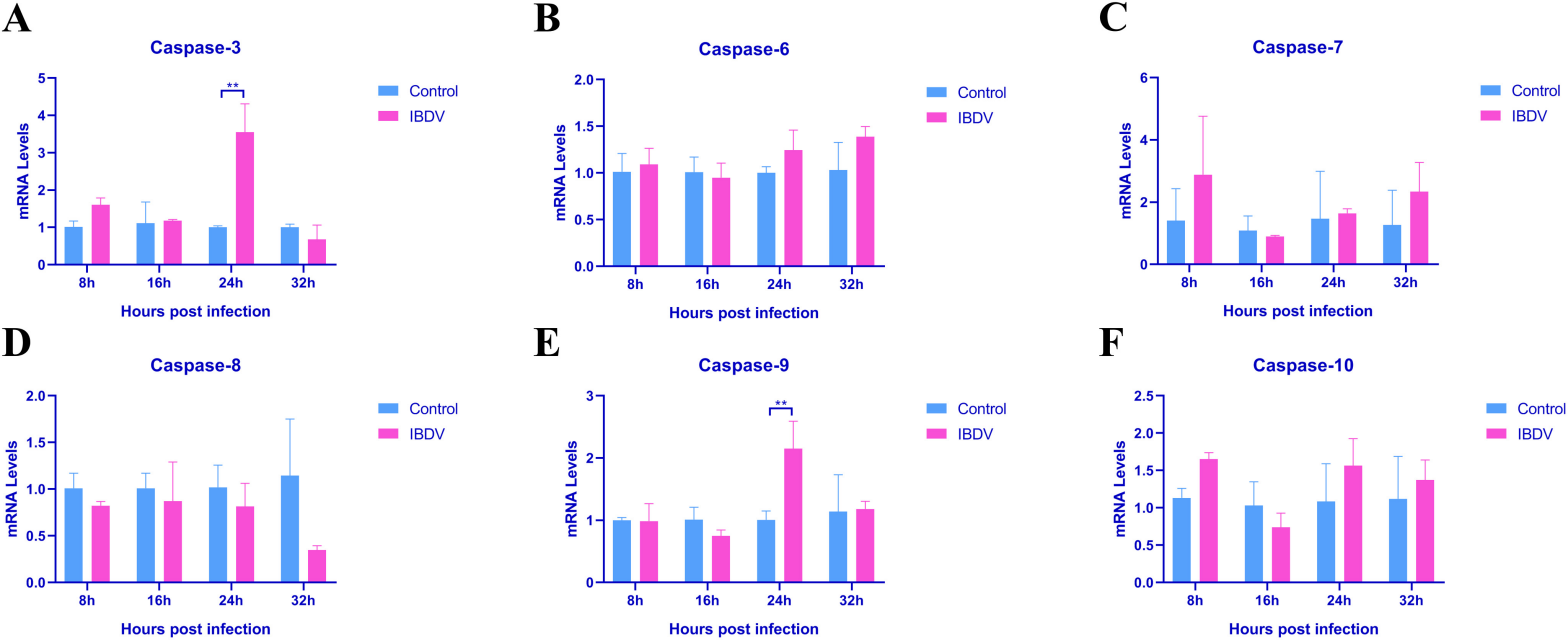
IBDV-caused apoptosis via mitochondrial pathway. **(A)** Caspase-3 levels. **(B)** Caspase-6 levels. **(C)** Caspase-7 levels. **(D)** Caspase-8 levels. **(E)** Caspase-9 levels. **(F)** Caspase-10 levels. RT-qPCR analysis was conducted on IBDV-infected DF-1 cells at 8, 16, 24, and 32 hpi, with uninfected control groups included for comparison. Results are presented as mean ± SD, analyzed using a two-sample Student’s *t*-test. *P < 0.05, **P < 0.01.

To further explore which Caspase plays a key role in apoptosis caused by IBDV infection, we further examined the expression levels of three initiator caspases (Caspase-8, Caspase-9, and Caspase-10) and three effector caspases (Caspase-3, Caspase-6, and Caspase-7) in IBDV-infected cell, using RT-qPCR. As shown in Figure 2, the mRNA expression levels of Caspase-3 and Caspase-9 exhibited significantly upregulated at 24 hpi as compared to uninfected control. In contrast, none of the other caspases displayed significant changes at any time point. These findings suggest that IBDV-induced apoptosis is closely associated with the intrinsic apoptotic pathway and caspase-3, caspase-9 might play important role.

### 3.3 Caspase-3 inhibitor Z-DEVD reduced the RNA and protein synthesis of IBDV in DF-1 cells

Given that effector Caspase-3 might play an important role in IBDV infection, and considering Caspase-3 as a downstream executor in the apoptotic pathway, the effect of Caspase-3 on the IBDV replication was further investigate. By using the Caspase-3 specific inhibitor Z-DEVD, and then IBDV was infected in DF-1 cell, RT-qPCR and Western blot analysis to assess the synthesis of IBDV RNA and protein, respectively.

**FIGURE 3:**
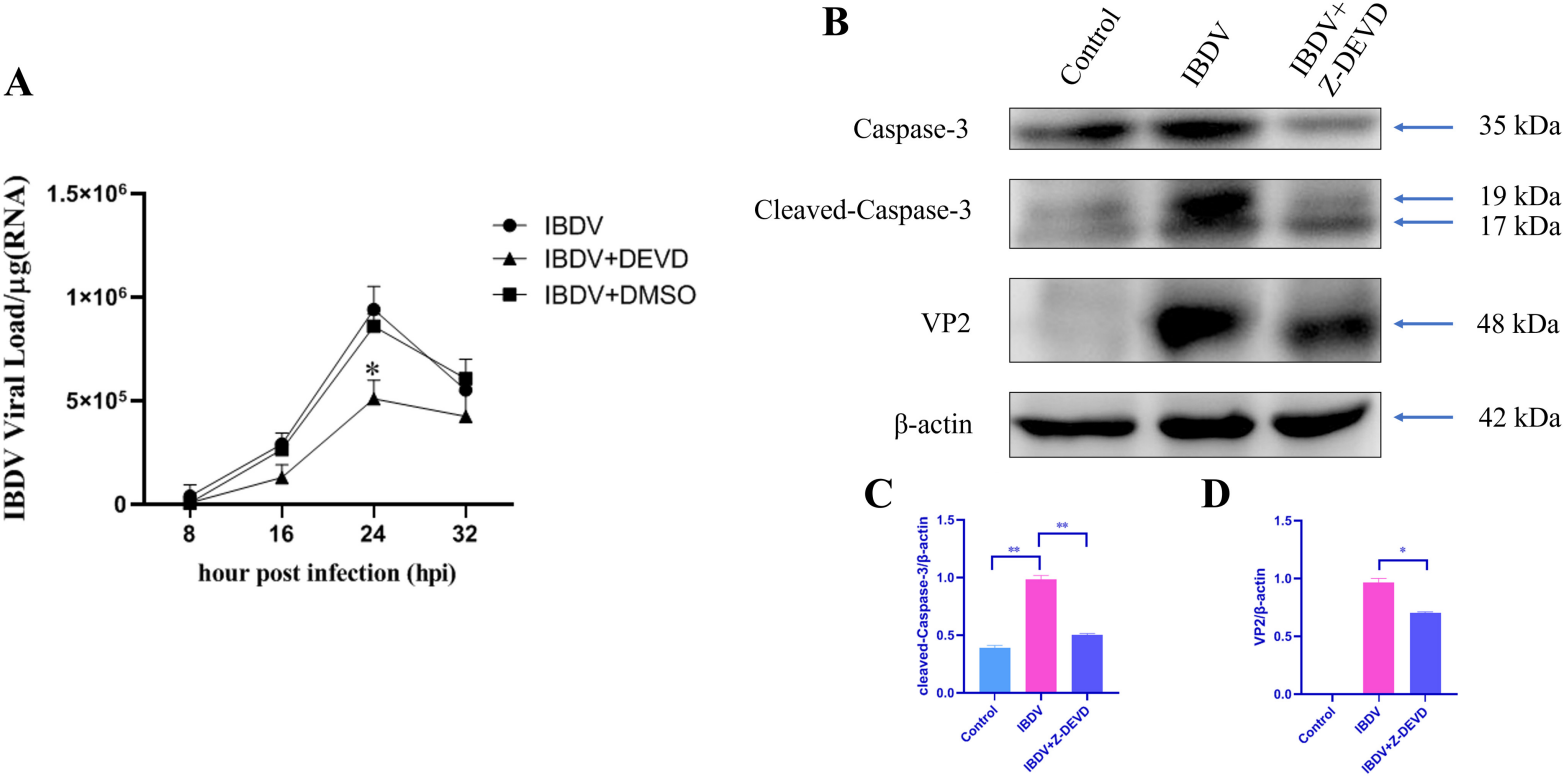
The caspase-3 inhibitor Z-DEVD reduces RNA and protein synthesis of IBDV in DF-1 cells. **(A)** IBDV-infected DF-1 cells were treated with control (no treatment), Z-DEVD (20 μM), or DMSO, and viral loads were measured at 8, 16, 24, and 32 hpi. **(B)** DF-1 cells were subjected to immunoblotting at 24 hpi after treatment with control, IBDV infection, or simultaneous IBDV infection and Z-DEVD treatment, using the indicated antibodies. β-actin was monitored as a loading control. **(C,D)** The gray-scale value ratios of target proteins to β-actin are presented as a measure of protein content. Results are expressed as mean ± SD, analyzed using a two-sample Student’s *t*-test. *P < 0.05, **P < 0.01.

The results from RT-qPCR indicated that IBDV RNA levels were lower in the Z-DEVD-treated group compared to the untreated group at all the detection time points, especially significantly lower at 24 hpi (Figure 3A). Consistently, the levels of the IBDV VP2 protein were also reduced in the Z-DEVD-treated group at this time point (Figures 3B,D). Moreover, we observed that IBDV infection significantly activated Caspase-3, while treatment with Z-DEVD notably decreased its activation (Figures 3B,C), which further confirmed the important role of caspase-3 in IBDV infection. These findings suggest that Caspase-3 inhibitor Z-DEVD treatment in DF-1 cells leads to a reduction in viral replication and synthesis.

### 3.4 Overexpression of caspase-3 promotes IBDV replication in DF-1 cells

**FIGURE 4:**
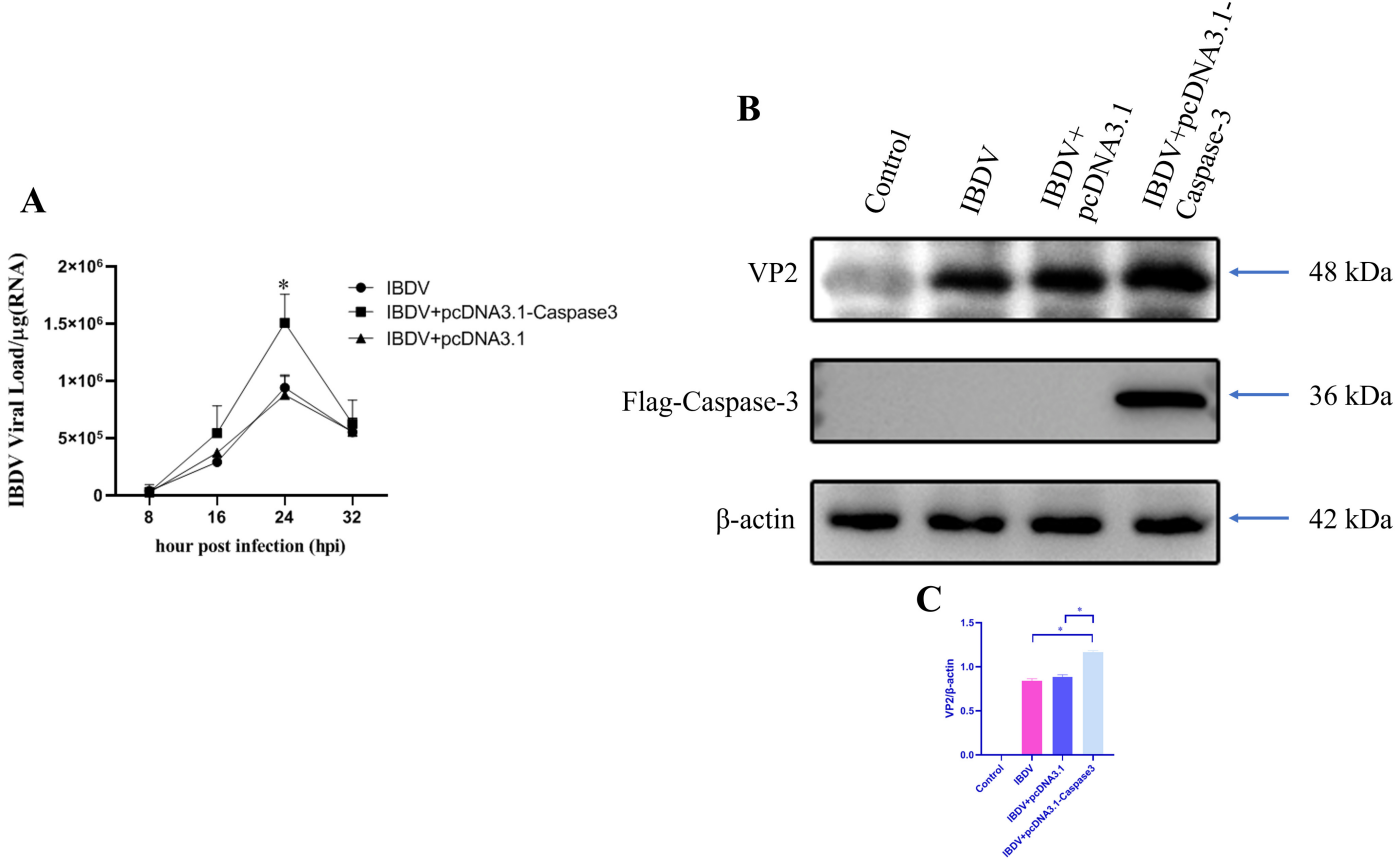
Overexpression of caspase-3 promotes IBDV replication. DF-1 cells were transfected with either pcDNA3.1 or pcDNA3.1-Caspase-3, and then infected with IBDV at 12 h post-transfection. **(A)** Viral loads were measured at 8, 16, 24, and 32 hpi. **(B)** Cells were collected at 24 hpi and analyzed by Western blotting. **(C)** The gray-scale value ratios of target proteins to β-actin are presented as a measure of protein content. Results are expressed as mean ± SD, analyzed using a two-sample Student’s *t*-test. *P < 0.05, **P < 0.01.

To further investigate the impact of Caspase-3 on IBDV replication, Caspase-3 was overexpressed by transfected DF-1 cells with pcDNA3.1-Caspase-3 plasmid and subsequently infected with IBDV. The results from RT-qPCR revealed that the viral load in the Caspase-3 overexpression group was significantly higher at 24 hpi compared to the group infected with IBDV alone (Figure 4A). Consistently, western-blotting analysis revealed that the IBDV VP2 protein was also significantly higher in the Caspase-3 overexpression group than that in the group infected with IBDV alone (Figures 4B,C). These findings further confirm that Caspase-3 plays a crucial role in the replication of IBDV.

### 3.5 Overexpression of caspase-3 inhibits the activation of IRF7 antiviral pathway and promotes apoptosis induced by IBDV

**FIGURE 5:**
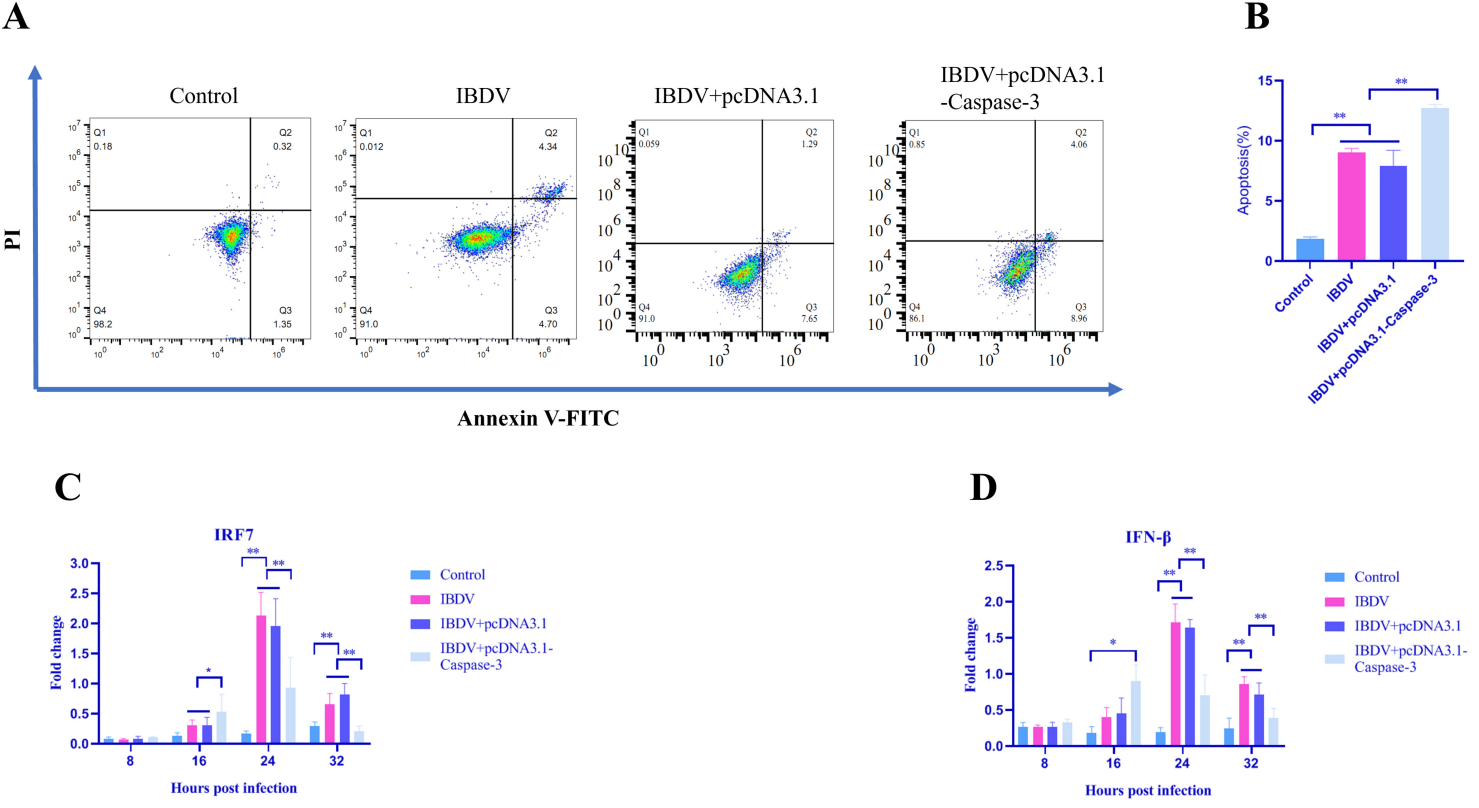
Overexpression of caspase-3 inhibits the activation of the IRF7 antiviral pathway and promotes apoptosis induced by IBDV. DF-1 cells were transfected with either pcDNA3.1 or pcDNA3.1-Caspase-3, and then infected with IBDV at 12 h post-transfection. **(A,B)** Annexin V-FITC and PI flow cytometry were performed to detect the number of apoptotic cells in DF-1 cells at 24 hpi. **(C,D)** RT-qPCR analysis was conducted at 8, 16, 24, and 32 hpi, with uninfected control groups included for comparison. Results are presented as mean ± SD, analyzed using a two-sample Student’s *t*-test. *P < 0.05, **P < 0.01.

The previous study indicates that the Caspase-3 promotes IBDV replication. Since chicken IRF7 has been demonstrated to restrict both RNA and DNA viral replication (e.g., NDV, AIV, and FPV) by upregulating IFN-β mRNA levels in chicken embryo fibroblasts (CEFs) (Cheng et al., 2019), together with our previously finding that IRF7 plays a crucial role in inhibiting IBDV replication in DF-1 cells (Wang Z. et al., 2024), the role of Caspase-3 in antiviral pathways in IBDV-infected cells were further evaluated. For this aim, Caspase-3 overexpression cells were established and followed by IBDV infection, then RT-qPCR was used to evaluate the changes in the mRNA levels of IRF7 and IFN-β,flow cytometry analysis was performed to evaluate the effects of the caspase-3 on the cell apoptosis. As shown in Figures 5A,B, the total apoptosis rates in cells induced by IBDV was higher in the Caspase-3 overexpression group compared to the group infected with IBDV alone. IBDV infection led to the upregulation of mRNA levels of IRF7 and IFN-β, particularly at 24 and 32 hpi. However, this upregulation was significantly inhibited following the overexpression of Caspase-3 (Figures 5C,D). These results suggested that Caspase-3 overexpression significantly impacts the activation of the IRF7 signaling pathway in response to IBDV infection, further, Caspase-3 promotes IBDV-induced cell apoptosis.

### 3.6 Caspase-3 cleaves IRF7

**FIGURE 6:**
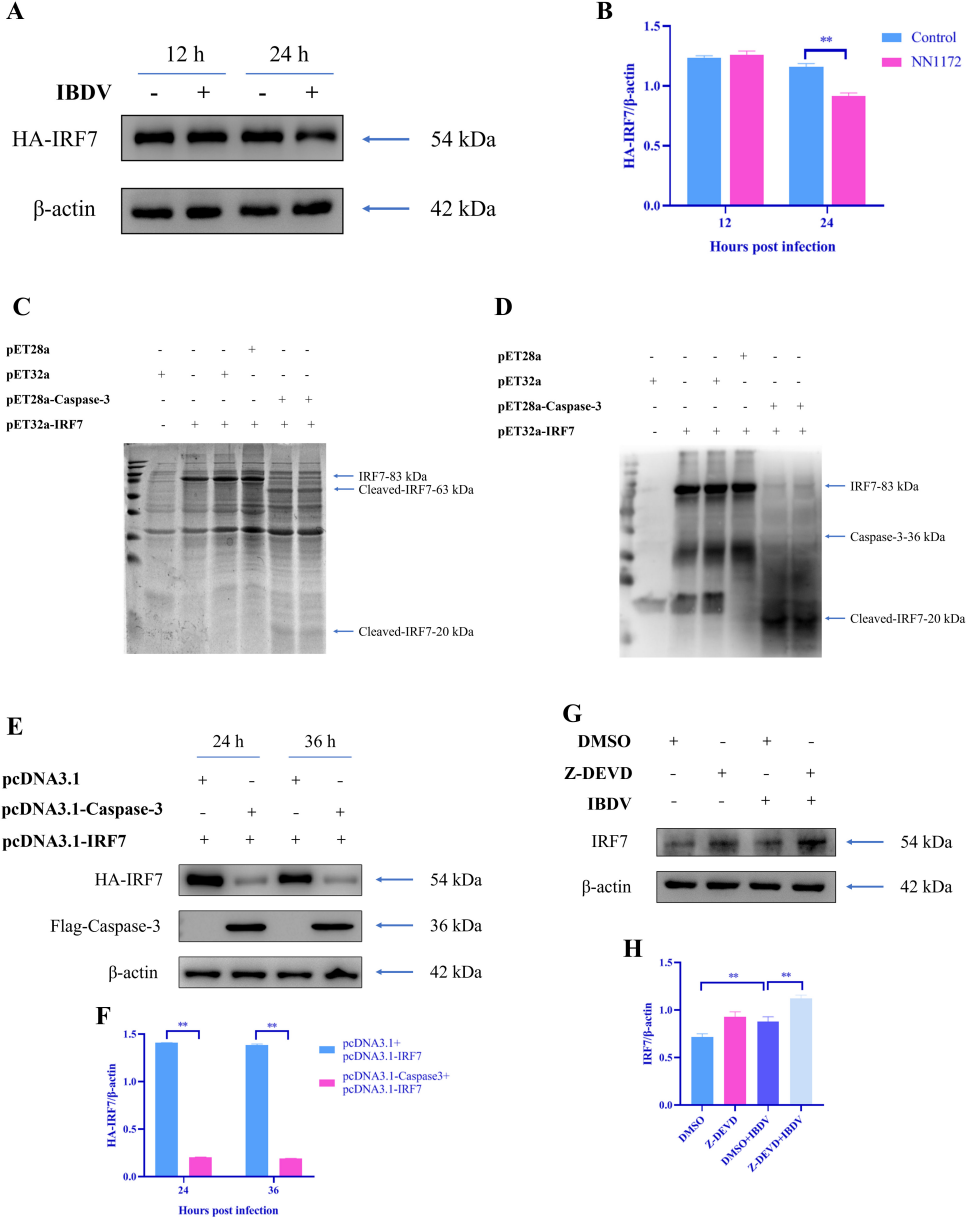
Caspase-3 cleaves IRF7. **(A,B)** DF-1 cells were transfected with the HA-tagged IRF7 plasmid and infected with IBDV at 12 h post-transfection. Cells were collected at 12 and 24 hpi for Western blot analysis. **(C,D)** The indicated plasmids were transformed, purified from BL21, and subjected to *in vitro* cleavage assays, followed by SDS-PAGE analysis and immunoblotting with the specified antibodies. **(E,F)** DF-1 cells were co-transfected with Flag-tagged Caspase-3 and HA-tagged IRF7 plasmids, and analyzed by Western blotting at 24 and 36 h post-transfection. **(G,H)** IBDV-infected DF-1 cells were treated with Z-DEVD (20 μM) for 24 h and the protein level of IRF7 was analyzed by Western blotting. Results are expressed as mean ± SD, analyzed using a two-sample Student’s *t*-test. *P < 0.05, **P < 0.01.

In the infection of swine acute diarrhea syndrome coronavirus (SADS-CoV), caspase-3 has been found to cleave interferon regulatory factor 3 (IRF3) to facilitate SADS-CoV replication (Wang X. et al., 2024). In our another study, IRF7 have been found to be degraded in IBDV-infected cell (Wang Z. et al., 2024). To further determine if the degradation of IRF7 is related to Caspase-3, the degradation of exogenous IRF7 in IBDV-infected cell were determined by employed a tagged IRF7 overexpression system followed by IBDV infection first. As expected, Western-blot analysis results showed that the IRF7 levels in the IBDV-infected groups were significantly lower than those in the uninfected groups at 24 hpi (Figures 6A,B). Then, an *in vitro* Caspase cleavage assay was conducted, where Caspase-3 and IRF7 prokaryotic expression products were co-incubated. SDS-PAGE analysis revealed the cleaved fragments of 63 kD and 20 kD (Figure 6C) as compared to the IRF7 control (83 kD). Western blotting confirmed that the 20 kD cleavage band reacted with His-tagged antibody (Figure 6D) which suggested that 20 kD cleavage band is part of His-tagged IRF7. Furthermore, when we co-overexpressed Caspase-3 and IRF7 in DF-1 cells, Western blotting analysis demonstrated that Caspase-3 efficiently cleaved IRF7 (Figures 6E,F). Meanwhile, we inhibited endogenous Caspase-3 activity using Z-DEVD and subsequently infected cells with IBDV. We observed that IBDV-induced degradation of endogenous IRF7 was significantly suppressed (Figures 6G,H).

### 3.7 IBDV VP3 interacts with caspase-3

**FIGURE 7:**
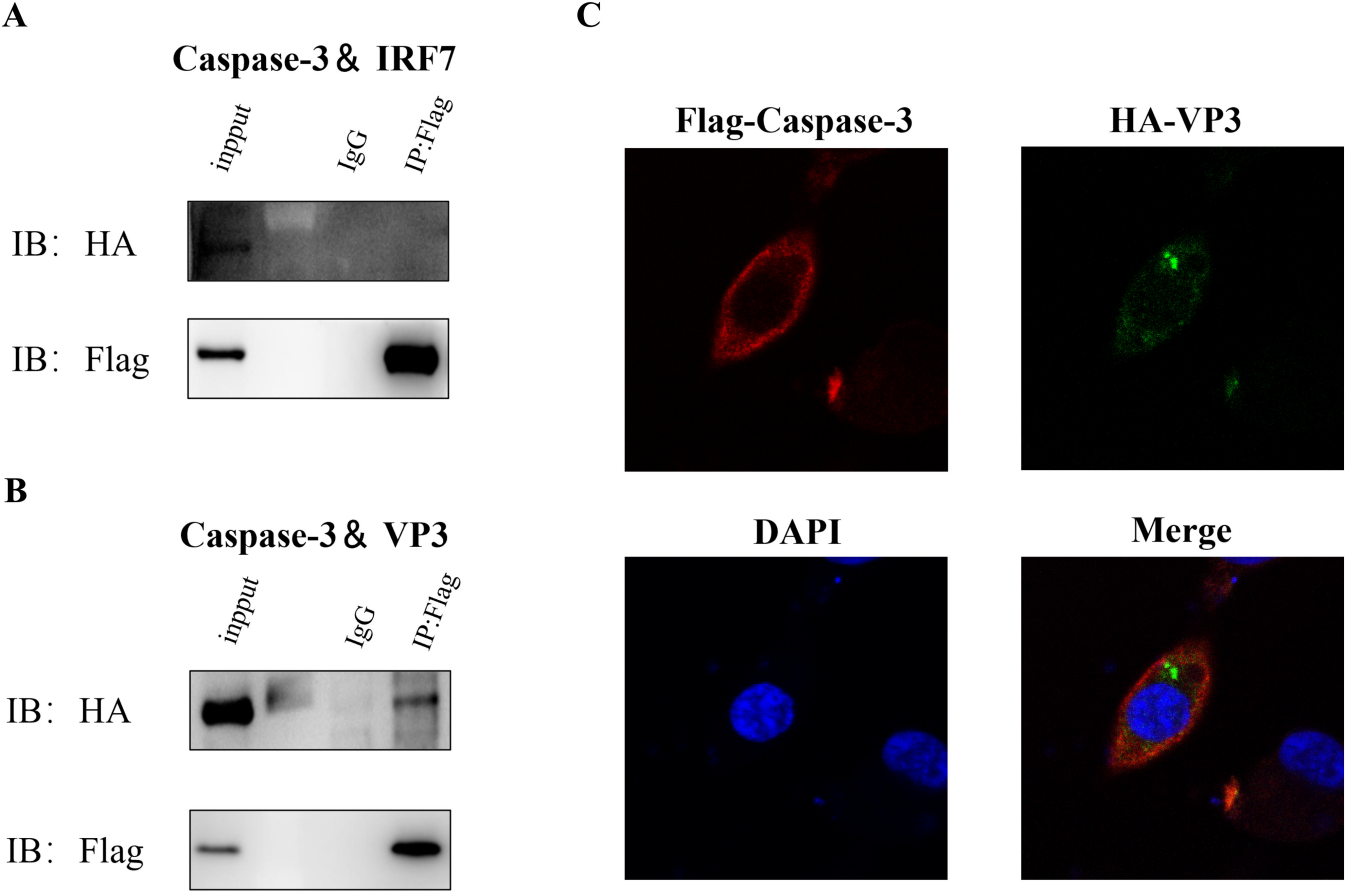
IBDV VP3 interacts with caspase-3. After co-transfecting DF-1 cells with corresponding plasmids, we harvested samples 48 h later. Cells were lysed and immunoprecipitation was performed using Flag-tag antibodies, followed by Western blotting detection. **(A)** Flag- Caspase-3 and HA-IRF7. **(B)** Flag- Caspase-3 and HA-VP3. **(C)** DF-1 cells transfected with p3 × Flag-CMV-14-Caspase-3 and pcDNA3.1-HA-VP3 were subjected to immunofluorescence staining with anti-Flag rabbit mAb and anti-HA mouse mAb.

The previous study revealed that Caspase-3 significantly cleaves IRF7, however, whether this cleavage depends on the direct interaction between the two protein is unknown. To investigate this interaction, we co-transfected DF-1 cells with the corresponding recombinant plasmids contain coding region of Caspase-3 (Flag-tag) or IRF7 (HA-tag) gene. Following transfection, we performed Co-IP using Flag-tag and HA-tag antibodies. However, as shown in Figure 7A, no direct interaction was observed between Caspase-3 and IRF7.

Our previous study demonstrated that IBDV VP3 directly interacts with IRF7 and significantly promotes IRF7 degradation to facilitate viral replication (Wang Z. et al., 2024). This finding led us to hypothesize a potential relationship between IBDV viral proteins and Caspase-3. Using the same approach, we performed Co-IP experiments. As shown in Figure 7B, the Flag-tag antibodies precipitated Flag-Caspase-3 and concurrently, HA-VP3, demonstrating an interaction between Caspase-3 protein and IBDV VP3 protein. To further validate the relationship between Caspase-3 and VP3, an immunofluorescence staining assay was conducted. We observed that exogenous Caspase-3 colocalized with exogenous viral protein VP3 in the cytoplasm of DF-1 cells (Figure 7C).

## 4 Discussion

In recent years, continuous mutations of IBDV field strains have led to the identification of an increasing number of gene reassortant strains, gene recombination strains, and Chinese novel variant strains (Fan et al., 2019; Wang et al., 2022a,b). IBDV remains a significant threat to the global poultry industry, as conventional vaccines fail to provide comprehensive protection (Fan et al., 2020), posing major challenges for IBD control. Following IBDV infection, intricate virus-host interactions occur. Thus, elucidating these interactions is critical for developing novel intervention strategies. Given the multifaceted role of caspases in modulating viral replication during infection (Best et al., 2003; Eléouët et al., 2000; Galvin and Husain, 2019; Méndez et al., 2004; Moody et al., 2007; Zhirnov et al., 1999), investigating the interplay between IBDV and caspases is essential to further unravel the pathogenic mechanisms of IBDV.

Apoptosis, the most well-characterized form of programmed cell death, can be triggered via intrinsic or extrinsic pathways (Galluzzi et al., 2012; Ichim and Tait, 2016). For the host, apoptosis serves as a critical defense mechanism against pathogen invasion by restricting viral replication and dissemination (Jorgensen et al., 2017). Previous studies have demonstrated a close association between IBDV infection and apoptosis, which relies on the activation of caspase-dependent signaling (Ojeda et al., 1997). Our findings reveal that both Z-VAD (a broad-spectrum caspase inhibitor) and Z-DEVD (a caspase-3-specific inhibitor) significantly suppressed IBDV-induced cell death and apoptosis. This aligns with observations by Xu et al. (2022), who reported that Z-VAD inhibited enterovirus A71 (EV-A71)-mediated apoptosis in Vero cells. Many viruses induce apoptosis in a caspase-dependent manner. For instance, Eléouët et al. (2000) demonstrated that broad-spectrum caspase inhibitors effectively blocked apoptosis triggered by transmissible gastroenteritis virus (TGEV), indicating that TGEV induces programmed cell death via a caspase-dependent pathway—consistent with our conclusions. Multiple IBDV-encoded proteins are implicated in apoptosis regulation. The VP2 protein was the first identified viral factor capable of inducing apoptosis (Lombardo et al., 1999). Busnadiego et al. (2012) reported that VP2 activates phosphorylation of protein kinase R (PKR), leading to host translational shutdown and subsequent apoptosis. Qin et al. (2017) further showed that VP2 triggers apoptosis by binding and degrading oral cancer overexpressed 1 (ORAOV1). Conversely, VP5 exhibits a dual role: Liu and Vakharia (2006) found that VP5 exerts anti-apoptotic effects during early infection, while Li et al. (2012) demonstrated that VP5 interacts with mitochondrial voltage-dependent anion channel 2 (VDAC2) to promote apoptosis. Additionally, Lin et al. (2015) revealed that receptor for activated C kinase 1 (RACK1) acts as an anti-apoptotic factor during IBDV infection by interacting with VDAC2 and VP5, thereby facilitating viral replication. Recent evidence indicates that VP3 possesses anti-apoptotic activity by suppressing PKR-mediated eIF2α phosphorylation and downstream apoptotic cascades, effectively counteracting VP2-induced apoptosis (Busnadiego et al., 2012). In summary, our results confirm that IBDV infection induces significant apoptosis, with multiple viral proteins intricately involved in modulating this process. These findings suggest that IBDV may exploit apoptotic pathways to counteract host defenses.

In this study, to systematically dissect the role of caspase-mediated apoptosis in IBDV pathogenesis, we employed a two-step pharmacological approach. First, we used the broad-spectrum caspase inhibitor Z-VAD to determine whether IBDV-induced cell death is generically dependent on caspase activation. A significant rescue effect by Z-VAD would implicate the caspase family as a whole in this process. Subsequently, to identify the key executor caspase responsible, we utilized Z-DEVD, a highly specific inhibitor of Caspase-3, the primary effector caspase in apoptotic cascades (Figure 1). This sequential strategy allowed us to progress from confirming the general involvement of caspases to pinpointing the specific contribution of Caspase-3, thereby providing a clear rationale for our subsequent mechanistic focus on this protease. As the primary executioner caspase, Caspase-3 cleaves multiple cellular substrates (Thornberry and Lazebnik, 1998), making it a focal point for investigating how IBDV exploits apoptosis to counteract host defenses. Conversely, Caspase-3 overexpression in DF-1 cells enhanced both viral replication and VP2 levels post-IBDV infection (Figure 4). These findings strongly suggest that Caspase-3 plays a potential role in IBDV replication. Supporting our observations, Duan et al. (2020) reported that IBDV infection epigenetically upregulates gga-miR-16-5p, which promotes viral replication by enhancing Bcl-2-mediated apoptosis. Notably, silencing either gga-miR-16-5p or Caspase-3 significantly suppressed IBDV replication—further corroborating our data. In conclusion, Caspase-3 serves as a critical host factor that IBDV exploits through a yet-undefined mechanism to facilitate its replication.

Our study demonstrated that overexpression of Caspase-3 in DF-1 cells enhanced IBDV-induced apoptosis and consequently promoted viral replication (Figure 4). This finding is consistent with Wurzer et al. (2003), who showed that inhibition or knockdown of Caspase-3 suppressed viral replication. Currently, three main mechanisms have been proposed to explain how viruses utilize Caspases to facilitate their replication: First, certain antiviral signaling proteins can be degraded by Caspases, thereby attenuating the induction of antiviral genes and counteracting host defense mechanisms. For instance, influenza A virus (IAV) strongly antagonizes the antiviral function of histone deacetylase 4 (HDAC4) through Caspase-mediated proteolysis (Galvin and Husain, 2019). Similarly, the coronavirus nucleocapsid (N) protein is cleaved by host Caspase-6, generating N fragments that interact with IRF3 and prevent its nuclear translocation, consequently inhibiting interferon responses and promoting viral replication (Eléouët et al., 2000). Second, some viruses exploit Caspase activity to cleave viral components, which may either promote or inhibit their own replication. The Aleutian mink disease parvovirus (AMDV) utilizes Caspase-3/-7 to cleave its major nonstructural protein (NS1), facilitating NS1 nuclear translocation and viral replication (Best et al., 2003). Conversely, the nucleocapsid protein of transmissible gastroenteritis coronavirus (TGEV) is targeted for destruction by host apoptotic machinery through cleavage by Caspase-6 and -7 (Eléouët et al., 2000). Human astrovirus employs Caspases to process the polyprotein precursor VP90 into VP70, which facilitates viral particle release (Méndez et al., 2004). Third, certain viruses activate Caspases to promote DNA replication and gene transcription. Human papillomavirus (HPV), for example, activates Caspase-3, -7, and -9 during differentiation, leading to cleavage and activation of its replication protein E1 to enhance viral production (Moody et al., 2007; Zhirnov et al., 1999). Based on these established mechanisms, we propose that IBDV may similarly exploit one of these strategies to promote its own replication. Concurrently, we examined IRF7 and IFN-β at the mRNA level. Results demonstrated that the Caspase-3-overexpressing group exhibited significantly lower expression of both IRF7 and IFN-β at 24 hpi compared to the IBDV-only infection group (Figures 5C,D), suggesting that Caspase-3 overexpression may suppress IFN-β-mediated antiviral responses by inhibiting IRF7 expression. Supporting this, Ouyang et al. (2018) observed upregulated MDA5 expression in IBDV-infected DT40 cells. Their work revealed that MDA5 overexpression activates the IFN-β and Mx promoters via an IRF7-dependent pathway, thereby restricting IBDV replication in DT40 cells. Our previous study further demonstrated that IBDV VP3 facilitates viral replication by degrading IRF7 (Wang Z. et al., 2024). Collectively, these findings implicate IRF7 as a critical host factor through which IBDV exploits Caspase-3 activity to enhance viral replication.

As previously discussed, Caspase-3 is a well-documented host factor that modulates viral replication through multiple mechanisms. However, its precise role in IBDV replication remains unclear. In this study, using an IRF7 overexpression system, we confirmed that IRF7 serves as a target of IBDV (Figures 6A,B), mirroring observations with other RNA viruses (NDV, AIV) (Cheng et al., 2019). Ning et al. (2019) reported that apoptotic caspases cleave cGAS, MAVS, and IRF3 in humans and mice to prevent excessive innate immune activation. While interferon regulatory factor 3 (IRF3) is the master regulator of virus-induced IFN production in mammals, chickens naturally lack IRF3 and instead utilize IRF7 to mediate IFN signaling in response to both DNA and RNA viral infections (Cheng et al., 2019). Intriguingly, our *in vitro* cleavage assays demonstrated that prokaryotically expressed Caspase-3 effectively degrades prokaryotically expressed IRF7 (Figures 6C,D). Similarly, exogenous IRF7 was significantly degraded in DF-1 cells when co-expressed with Caspase-3 (Figures 6E,F). To examine endogenous proteins, we inhibited intrinsic Caspase-3 activity using Z-DEVD and observed markedly reduced IRF7 degradation following IBDV infection (Figures 6G,H). These results collectively establish that Caspase-3 mediates IRF7 degradation. While our conclusions are strongly supported by the use of a specific pharmacological inhibitor (Z-DEVD) and overexpression studies, future work employing genetic knockdown or knockout of Caspase-3 in avian cells would provide an additional layer of definitive confirmation for its role in IBDV pathogenesis. Since our previous work confirmed that IRF7 strongly suppresses IBDV replication (Wang Z. et al., 2024), we conclude that IBDV exploits Caspase-3 activation to degrade IRF7 and thereby enhance viral replication. Notably, given the natural absence of RIG-I in chickens, Cheng et al. (2015) proposed an alternative MDA5-MAVS-STING-IRF7 signaling axis. This is particularly relevant as Ouyang et al. (2018) detected significant MDA5 upregulation in IBDV-infected DT40 cells. Furthermore, since Caspase-3 cleaves various upstream regulators of IRF3 (e.g., MAVS) in mammals (Ning et al., 2019), future studies should investigate whether Caspase-3 similarly processes IRF7-associated factors (e.g., MDA5, MAVS, or STING) to modulate innate immunity in avian hosts.

In this study, we observed that IBDV infection significantly activated Caspase-3, leading to Caspase-3-dependent degradation of IRF7. This finding prompted us to investigate the relationship between IBDV and Caspase-3. Co-immunoprecipitation (Co-IP) assays revealed a specific interaction between IBDV VP3 and Caspase-3 (Figure 7B), whereas no such interactions were detected for other viral proteins (VP1, VP2, VP4, or VP5). To further validate this interaction, we performed confocal microscopy, which demonstrated clear intracellular colocalization of VP3 and Caspase-3 (Figure 7C). Numerous studies have implicated Caspase-3 in viral pathogenesis, including infections by hepatitis B virus (Gottlob et al., 1998), herpes simplex virus (Kraft et al., 2006), and Japanese encephalitis virus (Yang et al., 2009). Thus, our results suggest that VP3 may modulate IBDV immune evasion mechanisms through its interaction with Caspase-3, underscoring the pivotal role of Caspase-3 in IBDV pathogenicity. Caspase-3 cleaves target proteins by specifically recognizing aspartate residues. While our data confirmed that Caspase-3 mediates IRF7 degradation *in vivo*, the underlying mechanism remained unclear. We hypothesized that direct Caspase-3–IRF7 binding might facilitate this process. However, Co-IP experiments ruled out a physical interaction between these two proteins (Figure 7A). Intriguingly, our prior work demonstrated a strong interaction between VP3 and IRF7 (Wang Z. et al., 2024). Although VP3 is dispensable for Caspase-3-mediated IRF7 degradation *in vitro*, we propose that VP3 may serve as a scaffold *in vivo*, bridging Caspase-3 and IRF7 to create a favorable microenvironment for degradation. This hypothesis offers a novel perspective for elucidating the Caspase-3–IRF7 regulatory axis.

Given that IRF7 is the central transcriptional activator of IFN-β in chickens, its degradation by Caspase-3 would be expected to impair the expression of a broad spectrum of interferon-stimulated genes (ISGs), which constitute the effector arm of the antiviral state. Meanwhile, our previous research also found that vvIBDV infection caused changes in the mRNA levels of ISGs (e.g., Mx, PKR, OAS) (Chen et al., 2022). Future studies profiling ISG expression (e.g., Mx, PKR, OAS) in this context will further delineate the comprehensive impact of this immune evasion mechanism on the host’s antiviral arsenal.

While our study elucidates a novel mechanism by which IBDV hijacks Caspase-3 to degrade IRF7 and subvert innate immunity, several limitations should be acknowledged. First, our work primarily focuses on caspase-dependent apoptosis in a chicken fibroblast cell line (DF-1). Although our data strongly support this pathway, we cannot exclude the potential contribution of other programmed cell death pathways (e.g., pyroptosis, necroptosis) in different cell types or *in vivo* contexts. Second, the findings are derived from *in vitro* experiments. The physiological relevance and therapeutic potential of targeting the Caspase-3/IRF7 axis need to be validated in future *in vivo* studies using chicken models. Furthermore, while we identified VP3 as an interactor of Caspase-3, the precise role of this interaction in facilitating IRF7 degradation or other viral functions warrants deeper investigation. Addressing these limitations in future work will provide a more comprehensive understanding of IBDV pathogenesis and advance the development of novel intervention strategies.

In summary, our study demonstrates that IBDV infection induces significant apoptosis and activates Caspase-3, which subsequently modulates viral replication through the IRF7 signaling pathway. More specifically, we reveal a novel mechanism whereby IBDV exploits Caspase-3-mediated degradation of IRF7 to facilitate viral propagation. These findings not only expand our understanding of IBDV’s innate immune evasion strategies but may also inform the development of more effective approaches for the clinical prevention and control of infectious bursal disease.

## Data Availability

The raw data supporting the conclusions of this article will be made available by the authors, without undue reservation.
