# Correction: Chicken caspase-3 promotes IBDV replication via the cleavage of IRF7

**DOI:** 10.3389/fmicb.2026.1819180

**Published:** 2026-03-10

**Authors:** Frontiers Production Office

**Affiliations:** Frontiers Media SA, Lausanne, Switzerland

**Keywords:** apoptosis, caspase-3, infectious bursal disease virus (IBDV), innate immunity, IRF7, viral replication

The affiliations of authors Hongjun Chen and Ping Wei were erroneously swapped during production. The correct author list and affiliations for the authors appear below.

Yang Chen^1†^, Jinnan Chen^1†^, Yanhua Xiang^1^, Simin Wei^1^, Minhui Zhao^1^, Kexuan Fu^1^, Yihai Li^1^, Hongjun Chen^3^, Ping Wei^2^ and Xiumiao He^1^^*^

^1^Guangxi Key Laboratory for Polysaccharide Materials and Modifications, School of Marine Sciences and Biotechnology, Guangxi Minzu University, Nanning, Guangxi, China

^2^College of Animal Science and Technology, Guangxi University, Nanning, Guangxi, China

^3^College of Veterinary Medicine, China Agricultural University, Beijing, China

The original version of this article has been updated.

